# Prevalence of the metabolic syndrome and associated factors among inpatients with severe mental illness in Botswana: a cross-sectional study

**DOI:** 10.1186/s12872-022-02939-5

**Published:** 2022-12-02

**Authors:** Billy M. Tsima, Philip Opondo, Mosepele Mosepele, Emang Mautle, Warren B. Bilker, Robert Gross

**Affiliations:** 1 Faculty of Medicine, University of Botswana, Gaborone, Botswana; 2 Sbrana Psychiatric Hospital, Lobatse, Botswana; 3 Perelman School of Medicine, University of Pennsylvania, Philadelphia, USA

**Keywords:** Metabolic syndrome, Severe mental illness, Cardiovascular diseases

## Abstract

**Introduction:**

The metabolic syndrome, a cluster of inter-related risk factors for cardiovascular diseases is highly prevalent among individuals with obesity and sedentary lifestyle. Chronic psychiatric disorders such as severe mental illness are associated with increased risk for cardiovascular diseases. We aimed to assess the prevalence and correlates of metabolic syndrome among inpatients with severe mental illness in a resource limited setting with high HIV prevalence.

**Methods:**

This was a cross-sectional study among adult inpatients at a referral psychiatric hospital in Botswana. We used convenience sampling to enrol participants available at the time of the study. The National Cholesterol Education Program Adult Treatment Panel-III (NCEP-ATP III) criteria was used to define the metabolic syndrome. Data were analysed using descriptive statistics as well as multiple logistic regression modelling.

**Results:**

A total of 137 participants were enrolled. Of these, 119 (87%) had complete data for the main analysis. The overall prevalence of metabolic syndrome was 22.6% (95% CI 15.9, 30.6) and did not differ significantly by gender or HIV status. Age was significantly associated with the risk of having the metabolic syndrome while gender, body mass index, HIV status, and days of moderate physical activity were not.

**Conclusion:**

There was a moderately high prevalence of metabolic syndrome. Thus, the management of individuals with severe mental illness in resource limited settings should include assessment of cardiovascular risk and target modifiable risk factors in this population. Consideration for the patient’s age should be made when rationalizing the limited resources available for assessing metabolic syndrome among patients with severe mental illness.

## Introduction

The metabolic syndrome (MetS) describes a cluster of risk factors for cardiovascular diseases and type 2 diabetes mellitus which include well recognised metabolic risk factors such as central obesity, dyslipidaemia, hypertension, glucose intolerance/diabetes and a prothromotic inflammatory state [1]. The prevalence of MetS is reportedly high among individuals with obesity and sedentary lifestyle [2]. Studies have shown that individual metabolic abnormalities of the MetS predict both type 2 diabetes and cardiovascular disease [3]. Therefore, the co-occurrence of these metabolic risk factors in an individual patient imposes additional risk. Furthermore, deaths from coronary heart diseases, cardiovascular diseases and all-cause mortality have been shown to be higher among patients with MetS compared to those without MetS [4, 5].

Severe mental illness (SMI) describes the presence of chronic psychiatric disorders that result in substantial functional impairment and typically includes the diagnosis of psychotic disorders, bipolar disorder, and recurrent major depressive disorder [6, 7]. People with SMI have worse physical health and life expectancy compared to the general population [7]. Interestingly, the increased mortality risk in people with SMI is mainly associated with cardiovascular diseases [8]. People with SMI are more likely to exhibit risk factors for cardiovascular diseases (CVD) such as obesity, dyslipidaemia, hypertension, and smoking relative to people without SMI [9]. Furthermore, second generation antipsychotics are widely used for acute and maintenance treatment of schizophrenia, acute mania, maintenance treatment in bipolar disorder and adjunctive therapy for major depressive disorder [10]. Although second generation antipsychotics have a clear therapeutic advantage over typical antipsychotics such as having a lower risk of extrapyramidal symptoms, they have been associated with metabolic abnormalities including an increased prevalence of metabolic syndrome [11]. MetS prevalence is 2–3 times greater in people with schizophrenia or bipolar disorder compared to the general population [12]. A growing body of evidence suggests SMI and MetS share certain pathological features, including hypothalamic-pituitary adrenal and mitochondrial dysfunction, neuroinflammation, common genetic links as well as epigenetic interactions [13–17]. Despite this well recognised risk for cardiovascular diseases among people with SMI, there is limited access to health care with less opportunity for cardiovascular risk screening and prevention for this group of patients than would be expected in a non-psychiatric population. The risk of having traditional CVD risk factors is also known to be high among people living with HIV (PLWHIV) [18, 19]. Additionally, PLWHIV are thought to be at an increased CVD risk secondary to HIV related inflammation and immune activation, or antiretroviral therapy induced dyslipidaemia, insulin resistance and glucose intolerance [20–23]. In general, people with SMI are at increased risk of HIV acquisition as well as cardiovascular diseases [24]. Compounding the problem, both mental illness [25] and HIV [26] confer a higher risk of tobacco use, which itself is a cardiovascular risk factor [27]. It follows that people with SMI and HIV infection are likely to carry a greater risk of cardiovascular diseases on account of the comorbid exposures. With the increasing number of PLWHIV surviving past the age of 50 years, the burden of chronic non-communicable diseases such as CVD is also increased. Therefore, CVD increasingly pose new challenges to healthcare systems globally [22, 28]. MetS can be used to identify patients who may benefit from interventions to reduce CVD risk [29]. Identifying factors associated with the risk for MetS among these individuals could help to target potential interventions to prevent it. This is more relevant to sub-Saharan Africa, where an epidemiologic transition has led to a dual burden of infectious and non-communicable diseases [30].

There is little published data reporting on the prevalence of MetS and its associated factors among individuals with SMI in resource limited settings despite increasing concerns of elevated risk of CVD among patients with SMI in high HIV burden settings [22, 23]. Patients with SMI admitted for inpatient management of their psychiatric illness in high HIV burden settings like Botswana provide an opportunity for screening for CVD. Unfortunately, this is not often done and these patient encounters tend to focus only on the psychiatric presentation even though this group of patients represents a potentially high risk for CVD. This lost opportunity to screen and treat a high risk group with significant multimorbidity which is engaged in care may contribute to the increasing burden of CVD in these settings. It is therefore important to evaluate the burden of CVD among inpatients with SMI in order to inform strategies needed to address this concern. We therefore aimed to assess the prevalence and correlates of MetS among inpatients with SMI in Botswana, a resource limited setting with high HIV prevalence. Our objectives were to (1) determine the prevalence of the MetS among inpatients with SMI at a psychiatric hospital in a resource constraint setting and (2) to identify clinical and sociodemographic correlates of the MetS among these patients.

## Methods

### Study design

We conducted a cross-sectional study to determine the prevalence and factors correlated with the MetS among patients with SMI in a psychiatric hospital in Botswana. The analysis was part of a study undertaken to determine whether HIV, mental illness, or both contribute to cardiovascular risk in patients with SMI. In the study, the primary outcome of carotid intima media thickness was measured to determine subclinical atherosclerosis as cardiovascular risk. Additionally, several variables known to be associated with cardiovascular risk were measured, thereby enabling an estimation of the MetS as described below. The analysis was restricted to observations with complete data to determine the MetS.

Study population and inclusion criteria.

The study population included adult inpatients (age 21 to 65 years) admitted to Sbrana Psychiatric Hospital (SPH) in Botswana, between February and August 2018 with a primary diagnosis of schizophrenia, schizoaffective disorder, schizophreniform disorder, psychotic disorder Not Otherwise Specified (NOS), bipolar disorder, or taking antipsychotic medications for at least 3 months. In addition, we included those admitted with major depression or anxiety disorder, or taking antidepressants/anxiolytics for at least 3 months. SPH is the national psychiatric referral hospital in Botswana and has a bed capacity of 300.

### Variables measured

We collected data on several variables that are known to be associated with cardiovascular risk. Weight was measured to the nearest 0.1 kg and height to the nearest 0.5 cm to obtain body mass index (BMI). Waist circumference (WC) was measured using a flexible tape measure to the nearest 0.5 cm measured on full exhalation at the level of the iliac crest. Smoking status was assessed through self-report. A participant’s affirmative response to the question “do you currently smoke any tobacco products, such as cigarettes, cigars or pipes?” was used to define a smoker. Venous blood was drawn for plasma total and high density lipoprotein (HDL) –cholesterol and triglycerides as well as low-density lipoprotein (LDL-C), measured at a single accredited (ISO 15,189) commercial laboratory. Blood samples were transported to the laboratory where plasma and red blood cells were separated and stored at -20^0^ Celcius. The plasma lipid profile was determined by enzyme colorimetric methods using an automatic chemistry analyzer machine (Abbott Architect, Abbott GmbH, Germany). Capillary blood glucose test by pinprick on fingertip was performed using a standardized automated point of care analyzer (ACCU-CHEK Performa, Roche, Germany). Level of physical activity as well as frequency of fruit and vegetable consumption were assessed using standard self-report instruments based on the WHO STEPwise approach to surveillance (STEPS) [31]. Blood pressure was measured using a digital sphygmomanometer and obtaining an average of three readings taken at least 3 min apart. The use of antihypertension medication was recorded. Patients with three or more of the following features: WC > 102 cm (males) or > 88 cm (females), triglycerides ≥ 1.7 mmol/L, high-density lipoprotein-cholesterol (HDL C) < 1.04 mmol/L (males) or < 1.30 mmol/L (females), BP ≥ 130/≥ 85 mmHg, and fasting glucose ≥ 6.1 mmol/L were considered to have the MetS in line with the National Cholesterol Education Program Adult Treatment Panel-III (NCEP-ATP III) criteria.

### Sample size and sampling

In the main study of which the current analysis is part of, a sample size of 137 was estimated to be required to detect a difference in cardiovascular risk between patients with SMI and HIV and those with SMI but without HIV in the study population. We used convenience sampling to enrol participants available during the data collection period. A study nurse approached inpatients meeting the inclusion criteria to recruit them for the study. A psychiatrist evaluated the patients’ capacity to provide informed consent and only those deemed competent were recruited. HIV testing was offered to all participants per Botswana national HIV guidelines which provide opt-out HIV testing in all medical care settings. Data were collected in a password protected RedCap database on a password protected computer.

### Data analysis

Comparisons of continuous variables were performed using Student’s t-test for variables with normal distribution or the Wilcoxon rank-sum test for variables not normally distributed. Pearson’s Chi-square test (or Fisher’s exact test where appropriate) was used for comparisons of categorical variables. The proportion of participants with the MetS was computed together with exact 95% confidence intervals.

Sociodemographic and clinical cardiovascular risk factors were evaluated as potential predictors for the MetS to compute unadjusted odds ratio (OR) and their exact 95% confidence interval (CI). All variables associated with MetS at a cut off *p*-value < 0.25 were included in multivariable logistic regression analyses controlling for age and sex as possible confounders to compute adjusted OR.

## Results

Out of a total of 137 participants enrolled, 119 (87%) had complete data for the main analysis. The sociodemographic, anthropometric and clinical characteristics of the participants stratified by sex are shown in Table 1. There was a slight predominance of males and nearly one quarter had HIV, which was more common among women than men, as has been reported previously in this setting [32].

**Table 1:** Sociodemographic, anthropometric and clinical characteristics of participants by sex

Variable	Overall(*n*=119)	Female(*n*=57)	Male(*n*=62)	*P* value
Age in years, median (IQR)	34(25.0, 43.0)	40(31,47)	28(24,37)	<0.001
Male sex, n (%)	62(52.1)	-	-	
HIV status, n (%)				<0.001
Positive	27 (23)	21 (37)	6(10)	
Negative	83 (72)	30 (53)	53 (86)	
Unknown	9 (5)	6 (11)	3 (5)	
Smoker, n (%)	43 (36)	5 (9)	38 (61)	<0.001
Cigarettes smoked per day, median (IQR)	5 (3,10)	3 (3,4)	6 (3,10)	0.40
^a^Type of antipsychotic medication used, n (%)				0.01
None	3 (3)	2 (4)	1 (2)	
1st generation	1 8(16)	3 (5)	15 (25)	
2nd generation	87 (75)	49 (88)	38 (63)	
Both	8 (7)	2 (4)	6 (10)	
Waist circumference in cm, mean (SD)	79.8 (15)	82.5 (18)	77.4 (11)	0.07
Hip circumference in cm, median(IQR)	87 (75, 96)	92 (77, 100)	84.5 (75, 92)	0.02
High Waist-hip ratio (>0.85 for females, >0.9 for males), n(%)	80 (67)	43 (75)	37 (60)	0.07
Weight in kilograms,	67 (58.1, 78)	66 (56.2, 81.5)	67.1 (60.8, 76)	0.87
Height in cm, median (IQR)	166.6 (161, 174)	161.5(157.5, 165.5)	170.9 (167.2, 178.2)	<0.001
BMI category, n (%)				0.01
Underweight (BMI <18)	8 (7)	2 (4)	6 (10)	
Normal (BMI 18-25)	61 (51)	24 (42)	37 (60)	
Overweight (BMI 26-30)	23 (19)	11 (19)	12 (19)	
Obese (BMI>30)	27 (23)	20 (35)	7 (11)	
SBP in mmHg, median (IQR)	121.7 (114,132)	123.3 (111.6, 133.7)	121.5 (114.3, 131.3)	0.52
DBP in mmHg, median (IQR)	82.3 (75.3, 89.7)	84.0 (76.6, 92.0)	81.7 (74.3, 87.3)	0.13
Components of the MetS, n(%)				
Central obesity	22 (19)	20 (36)	2 (3)	<0.0001
Elevated BP	58 (49)	30 (53)	28 (45)	0.42
Fasting glucose ≥6.1 mmol/L	46 (39)	23 (40)	23 (37)	0.72
Triglycerides ≥1.7 mmol/L	36 (30)	23 (40)	13 (21)	0.02
HDL-cholesterol, mmol/L (females <1.3 and males <1.0)	72 (61)	29 (51)	43 (69)	0.04
**Days/week engaged in moderate intensity activities, mean (SD)**	1.43(2.28)	1.32(2.01)	1.53(2.51)	0.61

^a^Patients with no current medications (n=11) were excluded

### Prevalence of metabolic syndrome

The overall prevalence of MetS was 18.5% (95% CI 11.4, 25.5) as shown in the figure. It was more common in females [24.6% (95% CI 13.4, 35.7)] than males [12.9% ( 95% CI 5.6, 21.2)], but this difference did not reach statistical significance, *p* = 0.1. The prevalence (95% CI) of MetS was slightly higher in PLWHIV at 18.5% (6.3, 38.1) than those without HIV at 14.5% (7.7, 23.9) although the difference was not statistically significant (p = 0.61).

### Factors associated with the metabolic syndrome

The univariable and multivariable logistic regression analyses for the association between different factors and the risk of participants having the metabolic syndrome is presented in Table 2. The variables gender, age, BMI category and HIV status met the inclusion criteria for the multivariable logistic regression at the set cut off point of *p*-value less than 0.25. The distribution of these variables is shown in Table 1. Only increasing age was associated with MetS both in univariable and multivariable analyses. Restricting the analysis to PLWHIV, only age and BMI category were associated with the MetS in the univariable but not the multivariable analysis.

**Table 2:** Univariable and multivariable logistic regression analysis of participants’ clinical and demographic characteristics and the metabolic syndrome

Characteristic	Unadjusted OR (95% CI)	*P* value	Adjusted OR (95% CI)	*P* value
Male sex	0.46 (0.17, 1.18)	0.11	0.99 (0.26, 3.82)	0.99
Age	1.10 (1.05, 1.16)	< 0.001	1.14 (1.07, 1.22)	0.00
BMI category	1.50 (0.91, 2.49)	0.11	1.39 (0.70, 2.78)	0.35
HIV	1.99 (0.79, 4.98)	0.14	1.10 (0.39, 3.12)	0.86
Days/week engaged in moderate intensity activities	0.80 (0.57, 1.11)	0.10	0.70 (0.49, 1.01)	0.06

^*BMI* Body Mass Index, *WHR* Waist hip ratio^

## Discussion

We found a moderately high prevalence of the MetS among patients with SMI admitted at a psychiatric hospital in Botswana. Our data indicate that nearly a fifth of our study participants met the NCEP-ATP III criteria for MetS. These findings are comparable with the recently reported MetS prevalence of 23.5% among patients with SMI attending a tertiary hospital in southwest Uganda [33], 24.5% among patients attending a psychiatric clinic in southern Ethiopia [34], and 23.2% among South African patients with SMI [35]. Data from resource limited settings outside the African continent, show similar prevalence rates at 21%, 22.8% and 29.4% in Mexico, Thailand and Brazil respectively [36–38]. In contrast, much higher prevalence rates have been found in many high income countries. For example, the prevalence of MetS among individuals with SMI in the United States, Australia and England has been estimated at 49.2%, 54% and 57% respectively [12, 39, 40].The geographical difference in the prevalence of MetS among individuals with SMI may be secondary to the influence of lifestyle and other environmental factors with or without genetic risk differences [17]. Differences in baseline MetS prevalence in the general population, which could be further heightened in SMI, may partially account for these geographical differences. It is also likely that the differences seen among different countries may be due to the different criteria used to define the metabolic syndrome. There are various criteria used for the diagnosis of MetS which although similar, will classify MetS differently. The criteria commonly used in the literature include the NCEP-ATP III [41], the modified NCEP-ATP III [42], the International Diabetes Federation (IDF) consensus statement [43], and the Joint Interim Statement [43] criteria. Additionally, the differences in treatment options, especially antipsychotics, available in different countries may also account for the geographical difference in MetS prevalence. Although second generation (atypical) antipsychotics are increasingly used in treating SMI given their therapeutic advantage of having lower incidence of extrapyramidal symptoms, the use of these medications is associated with development of metabolic abnormalities including MetS [44]. Since these medications are also more expensive than typical/first generation antipsychotics, they may not be as widely available in resource limited settings as much as they are in high income settings. In our study, more than three quarters of participants were on second generation antipsychotics at the time of the study. However, we did not determine the length of exposure to second generation antipsychotics and the specific type of second generation antipsychotic used due limitations in the quality of the clinical records. Our analysis is therefore limited by the inability to adjust for exposure to second generation antipsychotics in our model given that these medications are not a homogeneous group and known to have different propensities to cause weight gain [45].

The prevalence of MetS was higher among females with SMI compared to their male counterparts in our study. Although this difference appears to be clinically meaningful, it was not statistically significant. Similar findings have been reported in a study of comparable sample size from Spain among participants drawn from two community mental health centres [46] and elsewhere [40]. Our results are also consistent with results from previous meta-analyses that concluded that no gender differences in MetS prevalence existed [9, 17]. Other investigators have reported an apparent gender disparity in the prevalence of MetS, with most of these studies showing higher prevalence in women than men [33, 35, 47]. Interestingly, a cross-sectional analysis of over 4000 Taiwanese subjects under the age of 60 years showed a higher prevalence in men than women (22.3% vs. 15.8%, respectively). However, the male to female ratio was reversed after the age of 60 years [48].

Our multivariable logistic regression model revealed that age was significantly associated with the MetS. Other researchers have also reported an association between age and the MetS in populations from resource limited settings and similar age ranges [33–35]. This finding is consistent with results from studies conducted among the general population which have shown that the prevalence of MetS increases with age [49]. Furthermore, the risk for many of the conditions implicated in the pathophysiology of the MetS is known to increase with advancing age [3, 50]. Additionally, older patients with SMI may be exposed to antipsychotics for longer periods than younger patients and thus more likely to develop metabolic complications compared to their younger counterparts. It is therefore important to consider age as a prompt for assessing patients with SMI for MetS in resource limited settings.

We found no difference in the prevalence of MetS by HIV status. Similarly, no association between HIV and the prevalence of MetS was found in a recent cross-sectional study among patients with SMI in Uganda [33]. However, both studies may have been underpowered to detect a difference in prevalence of MetS by HIV status given the relatively small proportion of PLWHIV included in these analyses. Notably, other studies evaluating the prevalence of MetS among patients with SMI in high HIV burden resource limited settings have not accounted for HIV in their analyses. Data from non-psychiatric populations regarding the association between HIV and MetS have been inconsistent. However, the increased risk of MetS and its components among PLWHIV is well documented in the literature and has been attributed to HIV infection and antiretroviral therapy [30, 36]. Regrettably, low treatment rates for hypertension, dyslipidaemia and diabetes have been reported among patients with SMI. Undertreatment of hypercholesterolemia in PLWHIV has been documented in high HIV burden resource limited setting such as Botswana [51]. Efforts to address the discrepancy in treatment and outcome of cardiovascular diseases in people with SMI should ideally include assessment and management of cardiovascular risk factors such as the MetS in this vulnerable group. Determining the prevalence of MetS attributable to HIV or antiretroviral treatment is complicated by the background population and heterogeneity in the use of antiretrovirals in a cohort. Also, the diagnosis of MetS in this population can be more challenging if there is lipodystrophy and fat partitioning, so that abdominal obesity is not the predominant phenotype and waist circumference thresholds for the MetS are not met [52]. Interestingly, central obesity was the least prevalent component of the MetS in our study. Increasingly more researchers are advocating for the use of neck circumference instead of waist circumference to detect MetS [53, 54] since neck circumference correlates positively with traditional anthropometric indices such as BMI, waist circumference and waist-hip ratio [55]. The validity and clinical significance of neck circumference in evaluating MetS in high HIV burden resource limited African settings warrants exploration in future studies as it may be a simpler and reliable technique than waist circumference. Furthermore, there is an overrepresentation of common CVD risk factors such as physical inactivity and smoking in PLWHIV which heighten the risk of developing MetS [56].Therefore, in high HIV burden settings like Botswana, there should be a low threshold for evaluating patients for MetS and increased efforts at implementing risk reduction strategies such as smoking cessation interventions [57]. Other components of the MetS, namely, elevated BP, fasting blood glucose and triglycerides had comparable and relatively high prevalence while low HDL cholesterol had the highest prevalence. Among these, only blood pressure is routinely measured in clinical settings such as those of this study as it forms an integral part of nursing care to monitor vital signs of inpatients. A review of 39 internationally published studies indicated that rates of routine baseline screening for cholesterol and blood glucose were less than 50% [9]. Thus, the presence of these poorly monitored risk factors is likely to be missed. These results underscore the need for routine screening and monitoring of these components of the MetS as timely and sustained interventions targeting them have been shown to reduce the incidence of the MetS [58]. Importantly, opportunistic screening among patients with SMI admitted for care, can potentially aid in identifying those with the MetS and optimise their management [59].

### Limitations

Our study presents preliminary data on the MetS and its associated factors among patients with SMI in Botswana. However, the study has notable limitations. Firstly, our sample size was small owing to the challenges of recruiting participants with mental illness in empirical studies. Our small sample size limits the statistical power of our study and therefore larger studies are needed in similar settings to confirm our findings. Secondly, using convenience sampling may have introduced selection bias. Thirdly, we could not ascertain if participants adhered to fasting requirements for glucose and lipid measurements. Therefore, non-compliance with the NCEP-ATP III fasting guidelines may have led to an overestimation of the MetS. Additionally, our study was conducted among inpatients at a referral hospital thus limiting the generalizability of these findings. Future studies should determine to what extent out-patient risks for MetS are comparable with our findings from in-patient settings.

## Conclusion

The moderately high prevalence of MetS uncovered by our study underscores the need to screen and treat patients with SMI for cardiovascular risk factors. Thus, the management of individuals with SMI in resource limited settings should include an assessment of cardiovascular risk and target modifiable risk factors in this population. There is a need to improve access and streamline the care of patients with SMI in resource limited settings such as Botswana. Limited access to health care and a lack of coordinated care for individuals with mental illness are well recognized as contributing to the lower quality of physical health and reduced life expectancy among individuals with SMI [60]. Therefore improving access should be prioritized in these settings in order to reduce cardiovascular risk among individuals with SMI. Consideration of the patient’s age should be made when rationalizing the limited resources available for assessing MetS among patients with SMI.

## Data Availability

The datasets used for the study are available from the corresponding author on reasonable request.

## Abbreviations

MetS: Metabolic syndrome
CI: Confidence Interval
SMI: Severe mental illness
NCEP-ATP III: National Cholesterol Education Program Adult Treatment Panel-III
CVD: Cardiovascular diseases

## References

[1] Girman CJ, Dekker JM, Rhodes T, Nijpels G, Stehouwer CDA, Bouter LM (2005). An exploratory analysis of criteria for the metabolic syndrome and its prediction of long-term cardiovascular outcomes: the Hoorn study. Am J Epidemiol.

[2] Kivimäki M, Kuosma E, Ferrie JE, Luukkonen R, Nyberg ST, Alfredsson L (2017). Overweight, obesity, and risk of cardiometabolic multimorbidity: pooled analysis of individual-level data for 120 813 adults from 16 cohort studies from the USA and Europe. Lancet Public Health.

[3] Kassi E, Pervanidou P, Kaltsas G, Chrousos G (2011). Metabolic syndrome: definitions and controversies. BMC Med.

[4] Isomaa B, Almgren P, Tuomi T, Forsén B, Lahti K, Nissén M (2001). Cardiovascular morbidity and mortality associated with the metabolic syndrome. Diabetes Care.

[5] Malik S, Wong ND, Franklin SS, Kamath TV, L’Italien GJ, Pio JR (2004). Impact of the metabolic syndrome on mortality from coronary heart disease, cardiovascular disease, and all causes in United States adults. Circulation.

[6] Bhugra D (2005). The global prevalence of schizophrenia. PLoS Med.

[7] Miller BJ, Paschall CB, Svendsen DP (2006). Mortality and medical comorbidity among patients with serious mental illness. Psychiatr Serv Wash DC.

[8] Wilson PWF, D'Agostino RB, Parise H, Sullivan L, Meigs JB (2005). Metabolic syndrome as a precursor of cardiovascular disease and type 2 diabetes mellitus. Circulation..

[9] Mitchell AJ, Vancampfort D, De Herdt A, Yu W, De Hert M (2013). Is the prevalence of metabolic syndrome and metabolic abnormalities increased in early schizophrenia? A comparative meta-analysis of first episode, untreated and treated patients. Schizophr Bull.

[10] Meyer JM (2010). Antipsychotics and metabolics in the post-CATIE era. Curr Top Behav Neurosci.

[11] Meyer JM, Stahl SM (2009). The metabolic syndrome and schizophrenia. Acta Psychiatr Scand.

[12] McEvoy JP, Meyer JM, Goff DC, Nasrallah HA, Davis SM, Sullivan L (2005). Prevalence of the metabolic syndrome in patients with schizophrenia: baseline results from the Clinical Antipsychotic Trials of Intervention Effectiveness (CATIE) schizophrenia trial and comparison with national estimates from NHANES III. Schizophr Res..

[13] Correll CU, Joffe BI, Rosen LM, Sullivan TB, Joffe RT (2015). Cardiovascular and cerebrovascular risk factors and events associated with second-generation antipsychotic compared to antidepressant use in a non-elderly adult sample: results from a claims-based inception cohort study. World Psychiatry Off J World Psychiatr Assoc WPA.

[14] Manu P, Correll CU, Wampers M, Mitchell AJ, Probst M, Vancampfort D (2014). Markers of inflammation in schizophrenia: association vs. causation. World Psychiatry Off J World Psychiatr Assoc WPA.

[15] Nousen EK, Franco JG, Sullivan EL (2013). Unraveling the mechanisms responsible for the comorbidity between metabolic syndrome and mental health disorders. Neuroendocrinology.

[16] Rethorst CD, Bernstein I, Trivedi MH (2014). Inflammation, obesity, and metabolic syndrome in depression: analysis of the 2009–2010 National Health and Nutrition Examination Survey (NHANES). J Clin Psychiatry.

[17] Vancampfort D, Stubbs B, Mitchell AJ, De Hert M, Wampers M, Ward PB (2015). Risk of metabolic syndrome and its components in people with schizophrenia and related psychotic disorders, bipolar disorder and major depressive disorder: a systematic review and meta-analysis. World Psychiatry Off J World Psychiatr Assoc WPA.

[18] Obel N, Thomsen HF, Kronborg G, Larsen CS, Hildebrandt PR, Sørensen HT (2007). Ischemic heart disease in HIV-infected and HIV-uninfected individuals: a population-based cohort study. Clin Infect Dis Off Publ Infect Dis Soc Am.

[19] Shah RV, Murthy VL, Abbasi SA, Blankstein R, Kwong RY, Goldfine AB (2014). Visceral adiposity and the risk of metabolic syndrome across body mass index: the MESA Study. JACC Cardiovasc Imaging.

[20] Currier JS, Taylor A, Boyd F, Dezii CM, Kawabata H, Burtcel B (2003). Coronary heart disease in HIV-infected individuals. J Acquir Immune Defic Syndr 1999..

[21] Durand M, Sheehy O, Baril JG, Lelorier J, Tremblay CL (2011). Association between HIV infection, antiretroviral therapy, and risk of acute myocardial infarction: a cohort and nested case-control study using Québec’s public health insurance database. J Acquir Immune Defic Syndr..

[22] Masyuko SJ, Page ST, Kinuthia J, Osoti AO, Polyak SJ, Otieno FC (2020). Metabolic syndrome and 10-year cardiovascular risk among HIV-positive and HIV-negative adults: a cross-sectional study. Medicine (Baltimore)..

[23] Nou E, Lo J, Grinspoon SK (2016). Inflammation, immune activation, and cardiovascular disease in HIV. AIDS Lond Engl..

[24] De Santis JP (2009). HIV infection risk factors among male-to-female transgender persons: a review of the literature. J Assoc Nurses AIDS Care JANAC.

[25] Emul M, Kalelioglu T (2015). Etiology of cardiovascular disease in patients with schizophrenia: current perspectives. Neuropsychiatr Dis Treat.

[26] Mdodo R, Frazier EL, Dube SR, Mattson CL, Sutton MY, Brooks JT (2015). Cigarette smoking prevalence among adults with HIV compared with the general adult population in the United States: cross-sectional surveys. Ann Intern Med.

[27] Burns DM (2003). Epidemiology of smoking-induced cardiovascular disease. Prog Cardiovasc Dis.

[28] Shah ASV, Stelzle D, Lee KK, Beck EJ, Alam S, Clifford S (2018). Global burden of atherosclerotic cardiovascular disease in people living with HIV: systematic review and meta-analysis. Circulation..

[29] Verhaeghe N, De Maeseneer J, Maes L, Van Heeringen C, Annemans L (2011). Effectiveness and cost-effectiveness of lifestyle interventions on physical activity and eating habits in persons with severe mental disorders: a systematic review. Int J Behav Nutr Phys Act.

[30] Todowede OO, Mianda SZ, Sartorius B (2019). Prevalence of metabolic syndrome among HIV-positive and HIV-negative populations in sub-Saharan Africa-a systematic review and meta-analysis. Syst Rev.

[31] Riley L, Guthold R, Cowan M, Savin S, Bhatti L, Armstrong T (2016). The World Health Organization STEPwise approach to noncommunicable disease risk-factor surveillance: methods, challenges, and opportunities. Am J Public Health.

[32] Opondo PR, Ho-Foster AR, Ayugi J, Hatitchki B, Pumar M, Bilker WB (2018). HIV prevalence among hospitalized patients at the main psychiatric referral Hospital in Botswana. AIDS Behav.

[33] Agaba DC, Migisha R, Namayanja R, Katamba G, Lugobe HM, Aheisibwe H (2019). Prevalence and Associated Factors of Metabolic Syndrome among Patients with Severe Mental Illness Attending a Tertiary Hospital in Southwest Uganda. BioMed Res Int.

[34] Teshome T, Kassa DH, Hirigo AT (2020). Prevalence and Associated Factors of Metabolic Syndrome Among Patients with Severe Mental Illness at Hawassa, Southern-Ethiopia. Diabetes Metab Syndr Obes Targets Ther.

[35] Saloojee S, Burns JK, Motala AA (2016). Metabolic syndrome in South African patients with severe mental illness: prevalence and associated risk factors. PLoS ONE.

[36] Alvarez C, Salazar R, Galindez J, Rangel F, Castaãeda ML, Lopardo G (2010). Metabolic syndrome in HIV-infected patients receiving antiretroviral therapy in Latin America. Braz J Infect Dis Off Publ Braz Soc Infect Dis.

[37] Srisurapanont M, Likhitsathian S, Boonyanaruthee V, Charnsilp C, Jarusuraisin N (2007). Metabolic syndrome in Thai schizophrenic patients: a naturalistic one-year follow-up study. BMC Psychiatry.

[38] Teixeira PJR, Rocha FL (2007). The prevalence of metabolic syndrome among psychiatric inpatients in Brazil. Rev Bras Psiquiatr Sao Paulo Braz 1999.

[39] Gardner-Sood P, Lally J, Smith S, Atakan Z, Ismail K, Greenwood KE (2015). Cardiovascular risk factors and metabolic syndrome in people with established psychotic illnesses: baseline data from the IMPaCT randomized controlled trial. Psychol Med.

[40] John AP, Koloth R, Dragovic M, Lim SCB (2009). Prevalence of metabolic syndrome among Australians with severe mental illness. Med J Aust.

[41] Expert Panel on Detection, Evaluation, and Treatment of High Blood Cholesterol in Adults (2001). Executive summary of the third report of The National Cholesterol Education Program (NCEP) Expert panel on detection, evaluation, and treatment of high blood cholesterol in adults (Adult Treatment Panel III). JAMA.

[42] Alberti KGMM, Eckel RH, Grundy SM, Zimmet PZ, Cleeman JI, Donato KA (2009). Harmonizing the metabolic syndrome: a joint interim statement of the International Diabetes Federation Task Force on Epidemiology and Prevention; National Heart, Lung, and Blood Institute; American Heart Association; World Heart Federation; International Atherosclerosis Society; and International Association for the Study of Obesity. Circulation..

[43] Alberti KGMM, Zimmet P, Shaw J (2006). Metabolic syndrome–a new world-wide definition. A Consensus Statement from the International Diabetes Federation. Diabet Med J Br Diabet Assoc.

[44] Stahl SM, Mignon L, Meyer JM (2009). Which comes first: atypical antipsychotic treatment or cardiometabolic risk?. Acta Psychiatr Scand.

[45] Rummel-Kluge C, Komossa K, Schwarz S, Hunger H, Schmid F, Lobos CA (2010). Head-to-head comparisons of metabolic side effects of second generation antipsychotics in the treatment of schizophrenia: a systematic review and meta-analysis. Schizophr Res.

[46] Fernández Guijarro S, Miguel García C, Pomarol-Clotet E, Egea López EN, Burjales Martí MD, Rigol Cuadra MA (2020). Metabolic syndrome screening in people with severe mental illness: results from two spanish community mental health centers. J Am Psychiatr Nurses Assoc.

[47] de Caluwé L, van Buitenen N, Gelan PJ, Crunelle CL, Thomas R, Casseres S (2019). Prevalence of metabolic syndrome and its associated risk factors in an African-Caribbean population with severe mental illness. Psychiatry Res.

[48] Wang WS, Wahlqvist ML, Hsu CC, Chang HY, Chang WC, Chen CC (2012). Age- and gender-specific population attributable risks of metabolic disorders on all-cause and cardiovascular mortality in Taiwan. BMC Public Health..

[49] Grundy SM, Cleeman JI, Daniels SR, Donato KA, Eckel RH, Franklin BA (2005). Diagnosis and management of the metabolic syndrome: an American Heart Association/National Heart, Lung, and Blood Institute Scientific Statement. Circulation.

[50] Guarner-Lans V, Rubio-Ruiz ME, Pérez-Torres I, Baños de MacCarthy G (2011). Relation of aging and sex hormones to metabolic syndrome and cardiovascular disease. Exp Gerontol.

[51] Mosepele M, Letsatsi V, Mokgatlhe L, Hudson FP, Gross R (2017). Cholesterol screening and statin prescription is low among HIV-infected patients on protease-inhibitor regimens in Botswana. Open AIDS J.

[52] Samson SL, Garber AJ (2014). Metabolic syndrome. Endocrinol Metab Clin North Am.

[53] Cui T, Yan BH, Liu Z, Yang H, Gyan M, Ma YX. Neck circumference: A valuable anthropometric measurement to detect metabolic syndrome among different age groups in China. Diabetes Metab Res Rev. 2018;34(3):e2966. 10.1002/dmrr.2966 29144029

[54] Namazi N, Larijani B, Surkan PJ, Azadbakht L (2018). The association of neck circumference with risk of metabolic syndrome and its components in adults: a systematic review and meta-analysis. Nutr Metab Cardiovasc Dis NMCD.

[55] Mendes CG, Barbalho SM, Tofano RJ, Lopes G, Quesada KR, Detregiachi CRP (2021). Is neck circumference as reliable as waist circumference for determining metabolic syndrome?. Metab Syndr Relat Disord..

[56] Møller SP, Amare H, Christensen DL, Yilma D, Abdissa A, Friis H (2020). HIV and metabolic syndrome in an Ethiopian population. Ann Hum Biol.

[57] Tsima BM, Moedi P, Maunge J, Machangane K, Kgogwane M, Mudojwa T (2020). Feasibility of implementing a novel behavioural smoking cessation intervention amongst human immunodeficiency virus-infected smokers in a resource-limited setting: A single-arm pilot trial. South Afr J HIV Med.

[58] Joseph P, Leong D, McKee M, Anand SS, Schwalm JD, Teo K (2017). Reducing the global burden of cardiovascular disease, part 1: the epidemiology and risk factors. Circ Res.

[59] Sud D, Laughton E, McAskill R, Bradley E, Maidment I (2021). The role of pharmacy in the management of cardiometabolic risk, metabolic syndrome and related diseases in severe mental illness: a mixed-methods systematic literature review. Syst Rev.

[60] De Hert M, Dekker JM, Wood D, Kahl KG, Holt RIG, Möller HJ (2009). Cardiovascular disease and diabetes in people with severe mental illness position statement from the European Psychiatric Association (EPA), supported by the European Association for the Study of Diabetes (EASD) and the European Society of Cardiology (ESC). Eur Psychiatry J Assoc Eur Psychiatr.

